# Supplementary material for: Parallel Evolution of Sex-Linked Genes across XX/XY and ZZ/ZW Sex Chromosome Systems in the Frog Glandirana rugosa
Source: Genes (Basel). 2023 Jan 18;14(2):257. doi: 10.3390/genes14020257 (PMC9956060; doi:10.3390/genes14020257)
Supplement: Supplementary file 1 [file genes-14-00257-s001.zip › Supplementary materials (revised)/Figures S .pptx]

## Slide 1
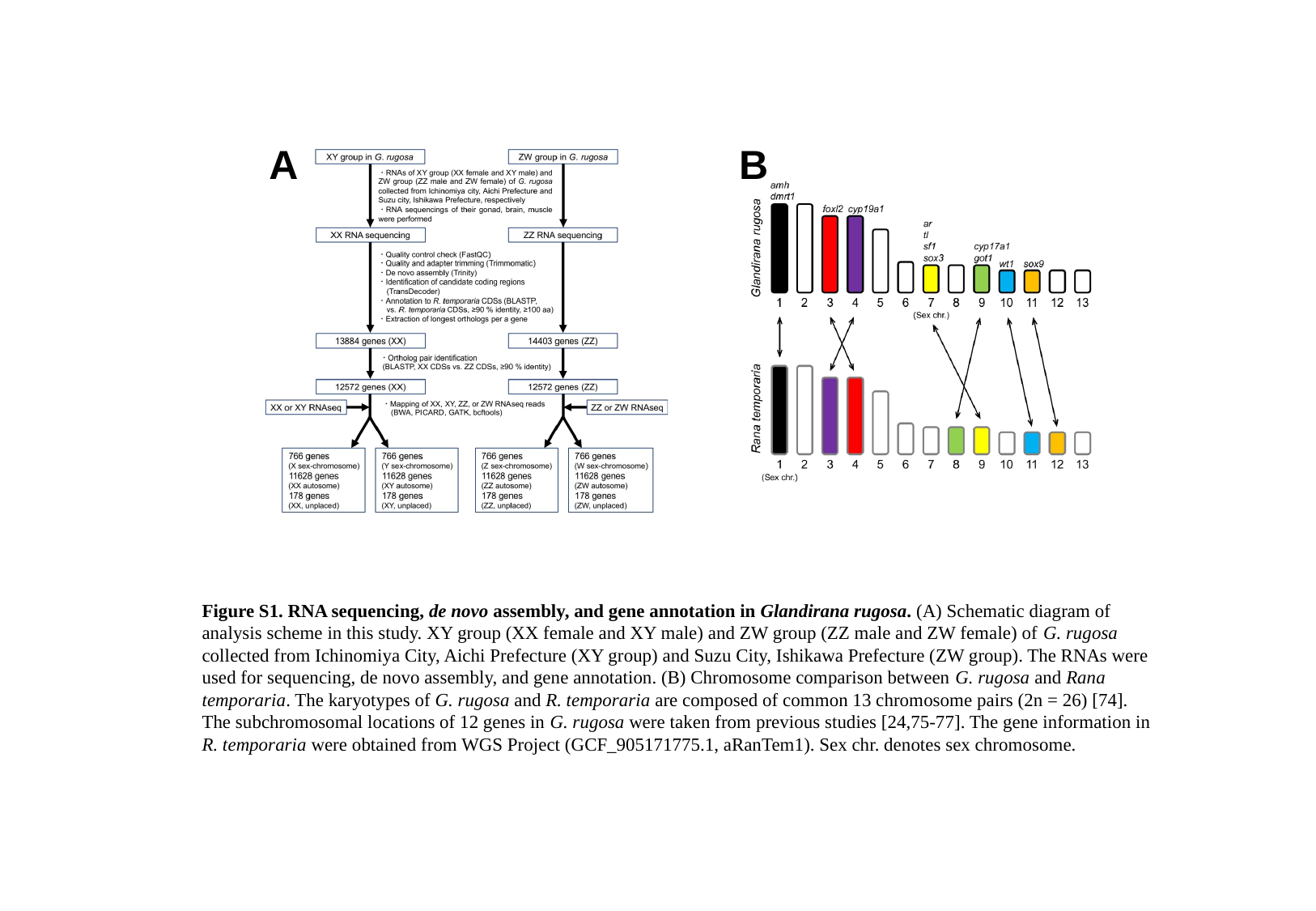

A
B
Figure S1. RNA sequencing, de novo assembly, and gene annotation in Glandirana rugosa. (A) Schematic diagram of analysis scheme in this study. XY group (XX female and XY male) and ZW group (ZZ male and ZW female) of G. rugosa collected from Ichinomiya City, Aichi Prefecture (XY group) and Suzu City, Ishikawa Prefecture (ZW group). The RNAs were used for sequencing, de novo assembly, and gene annotation. (B) Chromosome comparison between G. rugosa and Rana temporaria. The karyotypes of G. rugosa and R. temporaria are composed of common 13 chromosome pairs (2n = 26) [74]. The subchromosomal locations of 12 genes in G. rugosa were taken from previous studies [24,75-77]. The gene information in R. temporaria were obtained from WGS Project (GCF_905171775.1, aRanTem1). Sex chr. denotes sex chromosome.

## Slide 2
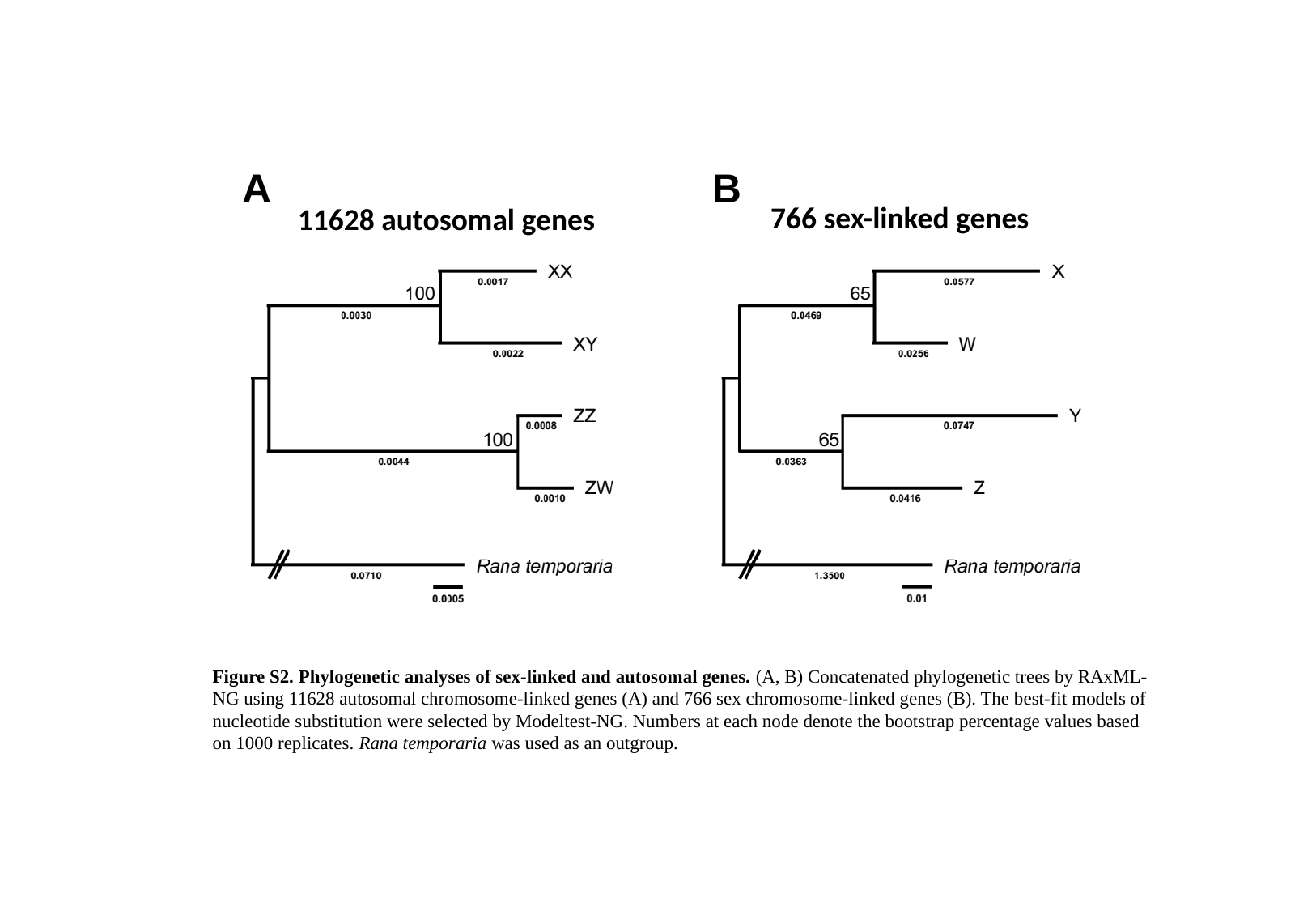

A
B
766 sex-linked genes
11628 autosomal genes
Figure S2. Phylogenetic analyses of sex-linked and autosomal genes. (A, B) Concatenated phylogenetic trees by RAxML-NG using 11628 autosomal chromosome-linked genes (A) and 766 sex chromosome-linked genes (B). The best-fit models of nucleotide substitution were selected by Modeltest-NG. Numbers at each node denote the bootstrap percentage values based on 1000 replicates. Rana temporaria was used as an outgroup.

## Slide 3
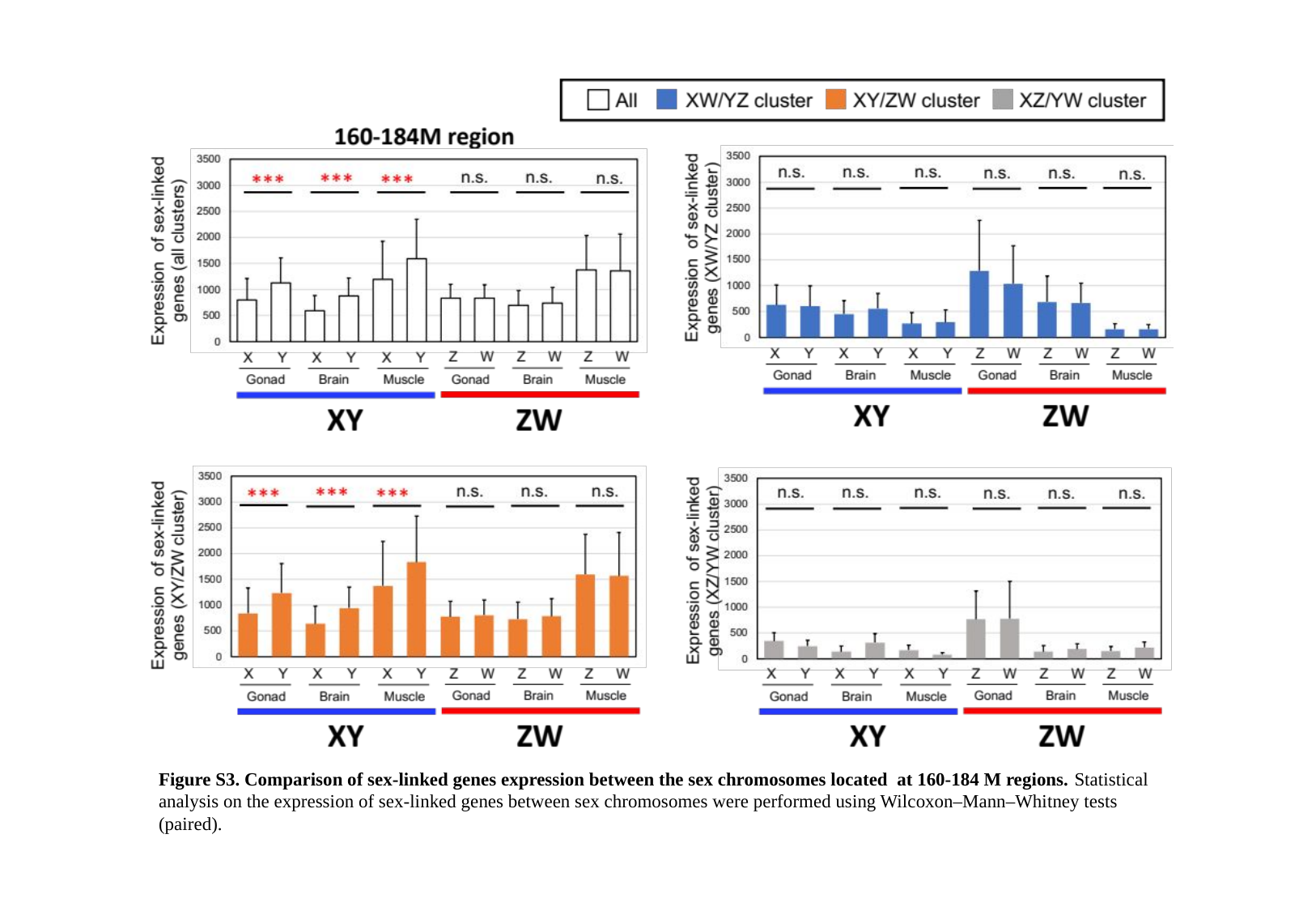

Figure S3. Comparison of sex-linked genes expression between the sex chromosomes located at 160-184 M regions. Statistical analysis on the expression of sex-linked genes between sex chromosomes were performed using Wilcoxon–Mann–Whitney tests (paired).

## Slide 4
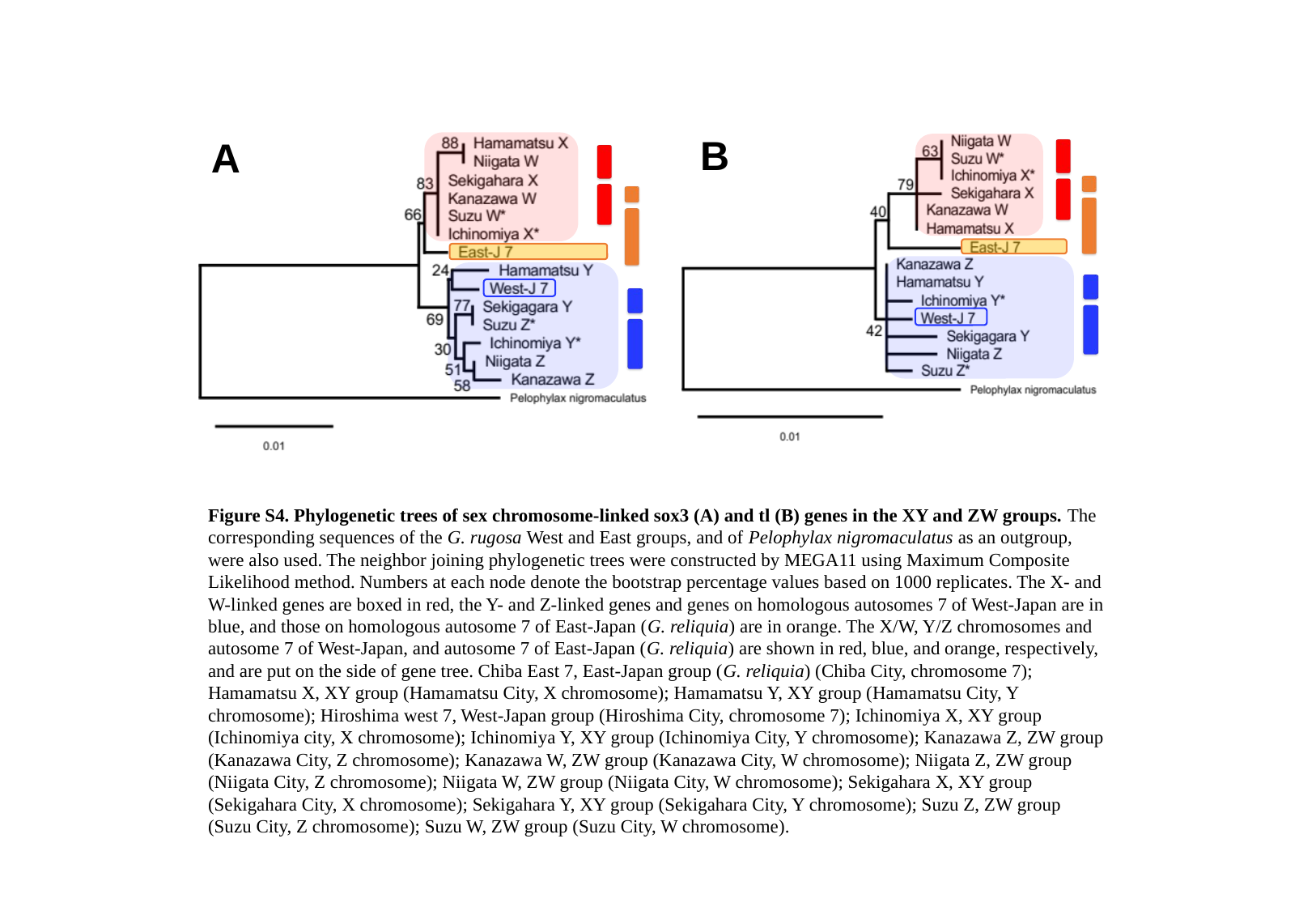

B
A
Figure S4. Phylogenetic trees of sex chromosome-linked sox3 (A) and tl (B) genes in the XY and ZW groups. The corresponding sequences of the G. rugosa West and East groups, and of Pelophylax nigromaculatus as an outgroup, were also used. The neighbor joining phylogenetic trees were constructed by MEGA11 using Maximum Composite Likelihood method. Numbers at each node denote the bootstrap percentage values based on 1000 replicates. The X- and W-linked genes are boxed in red, the Y- and Z-linked genes and genes on homologous autosomes 7 of West-Japan are in blue, and those on homologous autosome 7 of East-Japan (G. reliquia) are in orange. The X/W, Y/Z chromosomes and autosome 7 of West-Japan, and autosome 7 of East-Japan (G. reliquia) are shown in red, blue, and orange, respectively, and are put on the side of gene tree. Chiba East 7, East-Japan group (G. reliquia) (Chiba City, chromosome 7); Hamamatsu X, XY group (Hamamatsu City, X chromosome); Hamamatsu Y, XY group (Hamamatsu City, Y chromosome); Hiroshima west 7, West-Japan group (Hiroshima City, chromosome 7); Ichinomiya X, XY group (Ichinomiya city, X chromosome); Ichinomiya Y, XY group (Ichinomiya City, Y chromosome); Kanazawa Z, ZW group (Kanazawa City, Z chromosome); Kanazawa W, ZW group (Kanazawa City, W chromosome); Niigata Z, ZW group (Niigata City, Z chromosome); Niigata W, ZW group (Niigata City, W chromosome); Sekigahara X, XY group (Sekigahara City, X chromosome); Sekigahara Y, XY group (Sekigahara City, Y chromosome); Suzu Z, ZW group (Suzu City, Z chromosome); Suzu W, ZW group (Suzu City, W chromosome).

## Slide 5
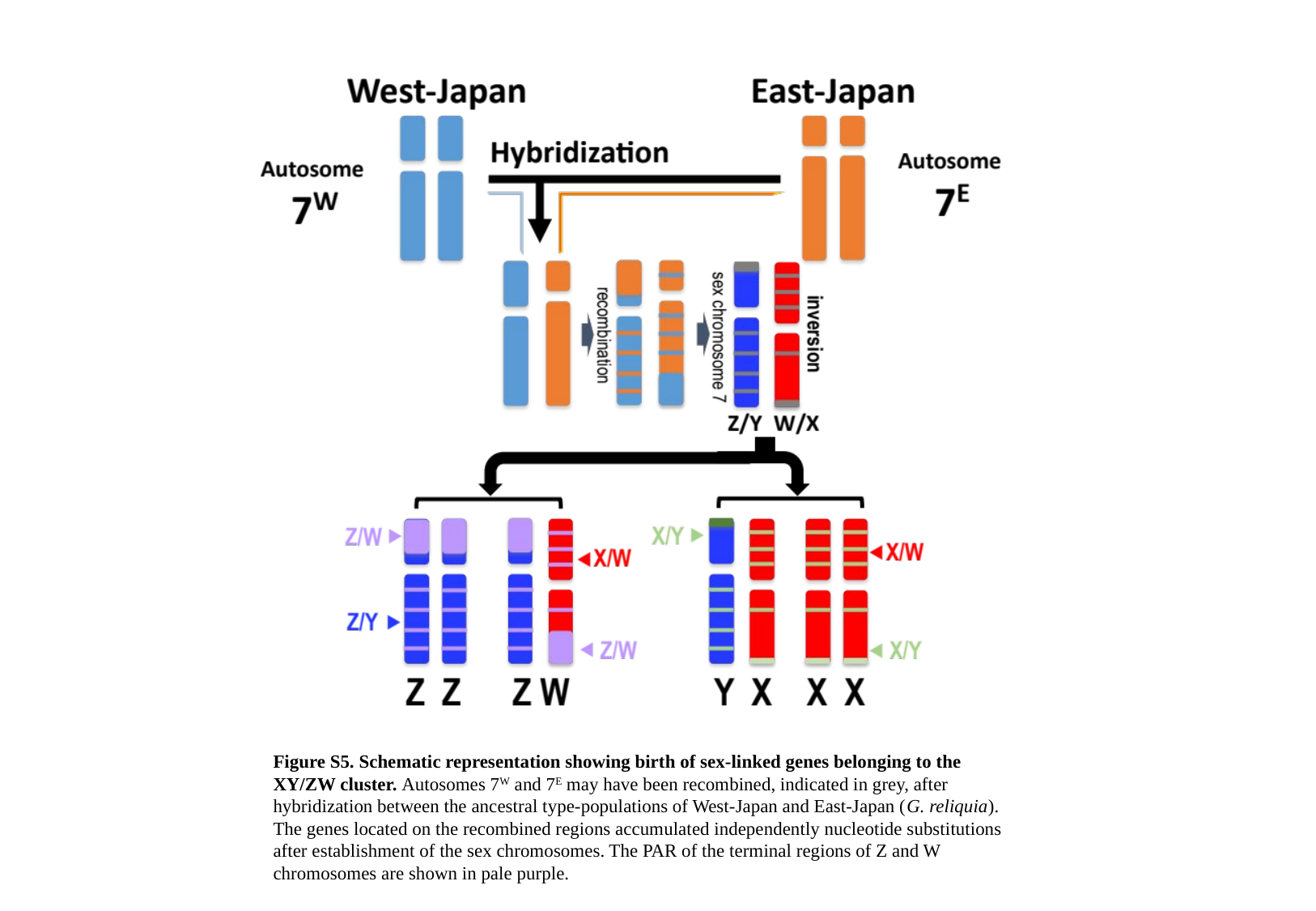

Figure S5. Schematic representation showing birth of sex-linked genes belonging to the XY/ZW cluster. Autosomes 7W and 7E may have been recombined, indicated in grey, after hybridization between the ancestral type-populations of West-Japan and East-Japan (G. reliquia). The genes located on the recombined regions accumulated independently nucleotide substitutions after establishment of the sex chromosomes. The PAR of the terminal regions of Z and W chromosomes are shown in pale purple.

## Slide 6
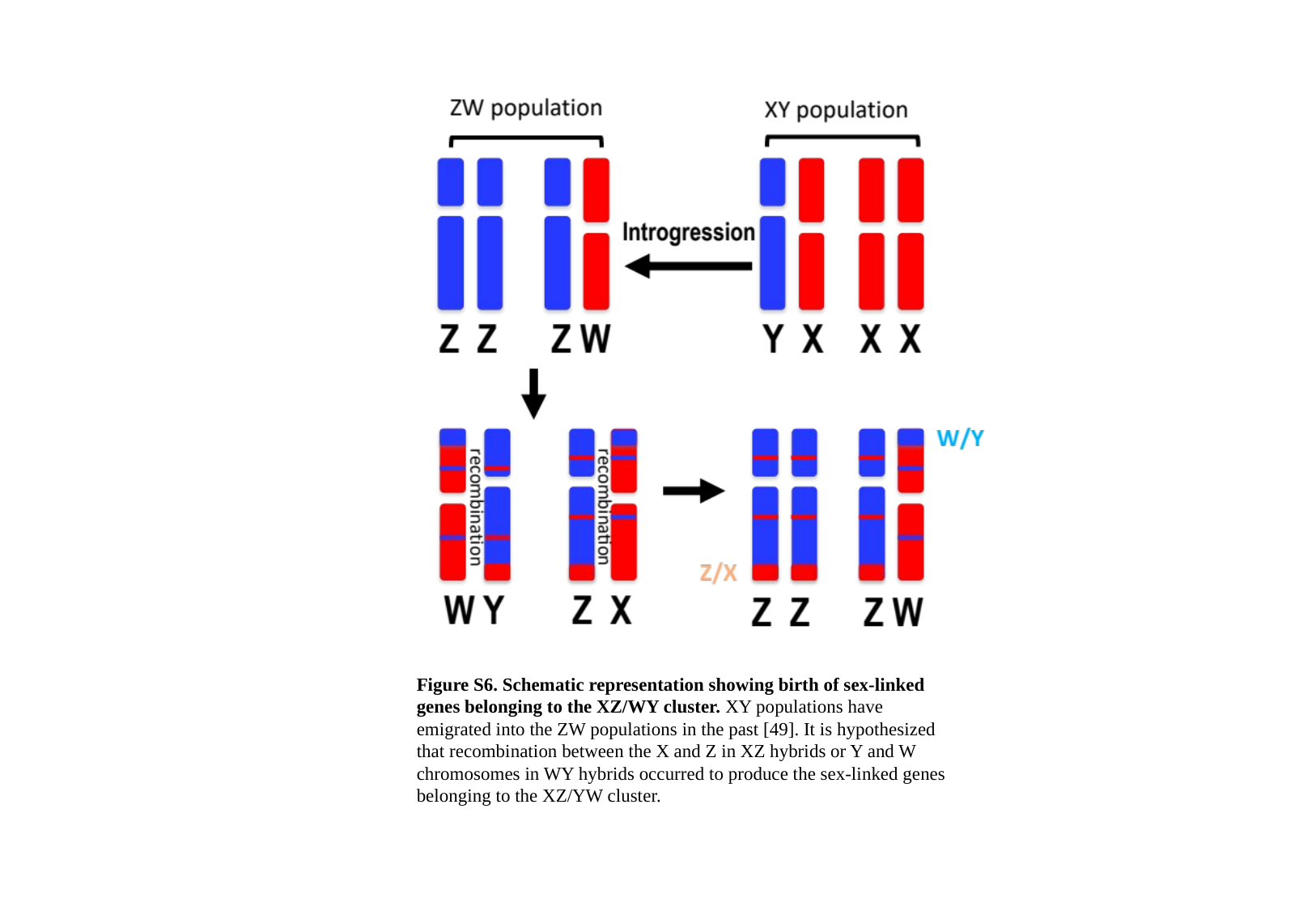

Figure S6. Schematic representation showing birth of sex-linked genes belonging to the XZ/WY cluster. XY populations have emigrated into the ZW populations in the past [49]. It is hypothesized that recombination between the X and Z in XZ hybrids or Y and W chromosomes in WY hybrids occurred to produce the sex-linked genes belonging to the XZ/YW cluster.

## Slide 7
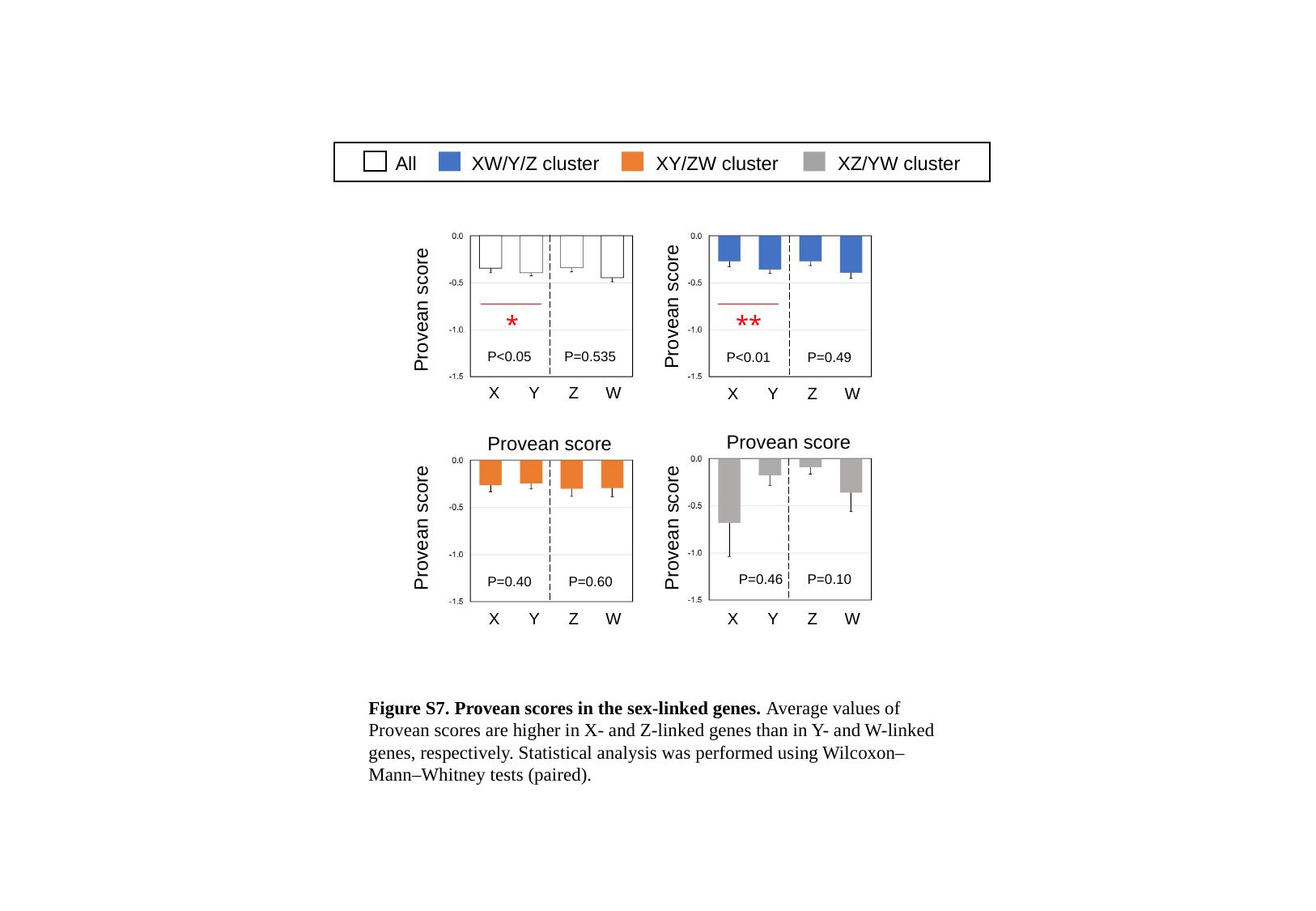

All
XW/Y/Z cluster
XY/ZW cluster
XZ/YW cluster
Provean score
Provean score
 **
 *
P<0.05
P=0.535
P<0.01
P=0.49
X
Y
Z
W
X
Y
Z
W
Provean score
Provean score
Provean score
Provean score
P=0.46
P=0.10
P=0.40
P=0.60
X
Y
Z
W
X
Y
Z
W
Figure S7. Provean scores in the sex-linked genes. Average values of Provean scores are higher in X- and Z-linked genes than in Y- and W-linked genes, respectively. Statistical analysis was performed using Wilcoxon–Mann–Whitney tests (paired).

## Slide 8
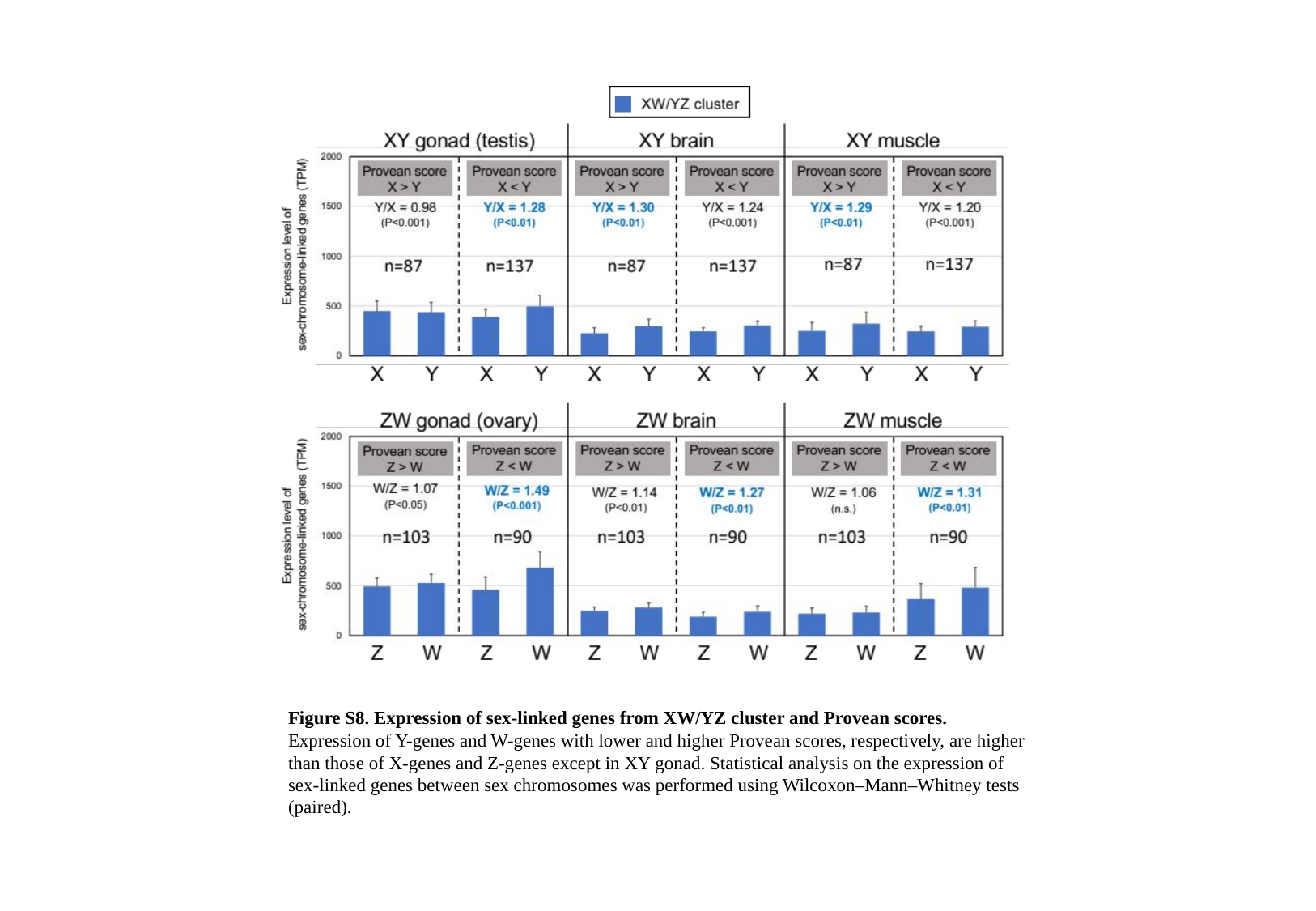

Figure S8. Expression of sex-linked genes from XW/YZ cluster and Provean scores. Expression of Y-genes and W-genes with lower and higher Provean scores, respectively, are higher than those of X-genes and Z-genes except in XY gonad. Statistical analysis on the expression of sex-linked genes between sex chromosomes was performed using Wilcoxon–Mann–Whitney tests (paired).

## Slide 9
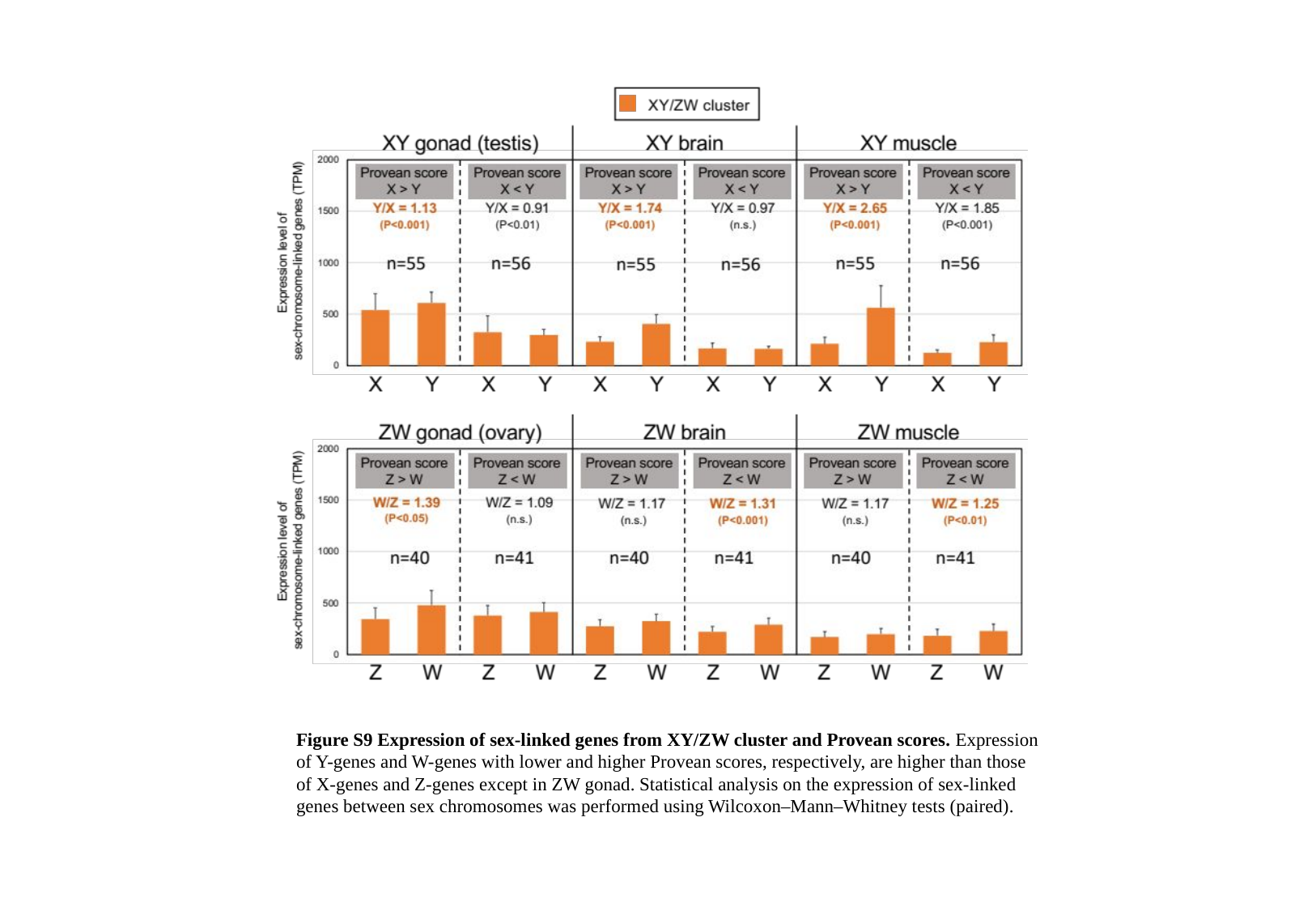

Figure S9 Expression of sex-linked genes from XY/ZW cluster and Provean scores. Expression of Y-genes and W-genes with lower and higher Provean scores, respectively, are higher than those of X-genes and Z-genes except in ZW gonad. Statistical analysis on the expression of sex-linked genes between sex chromosomes was performed using Wilcoxon–Mann–Whitney tests (paired).

## Slide 10
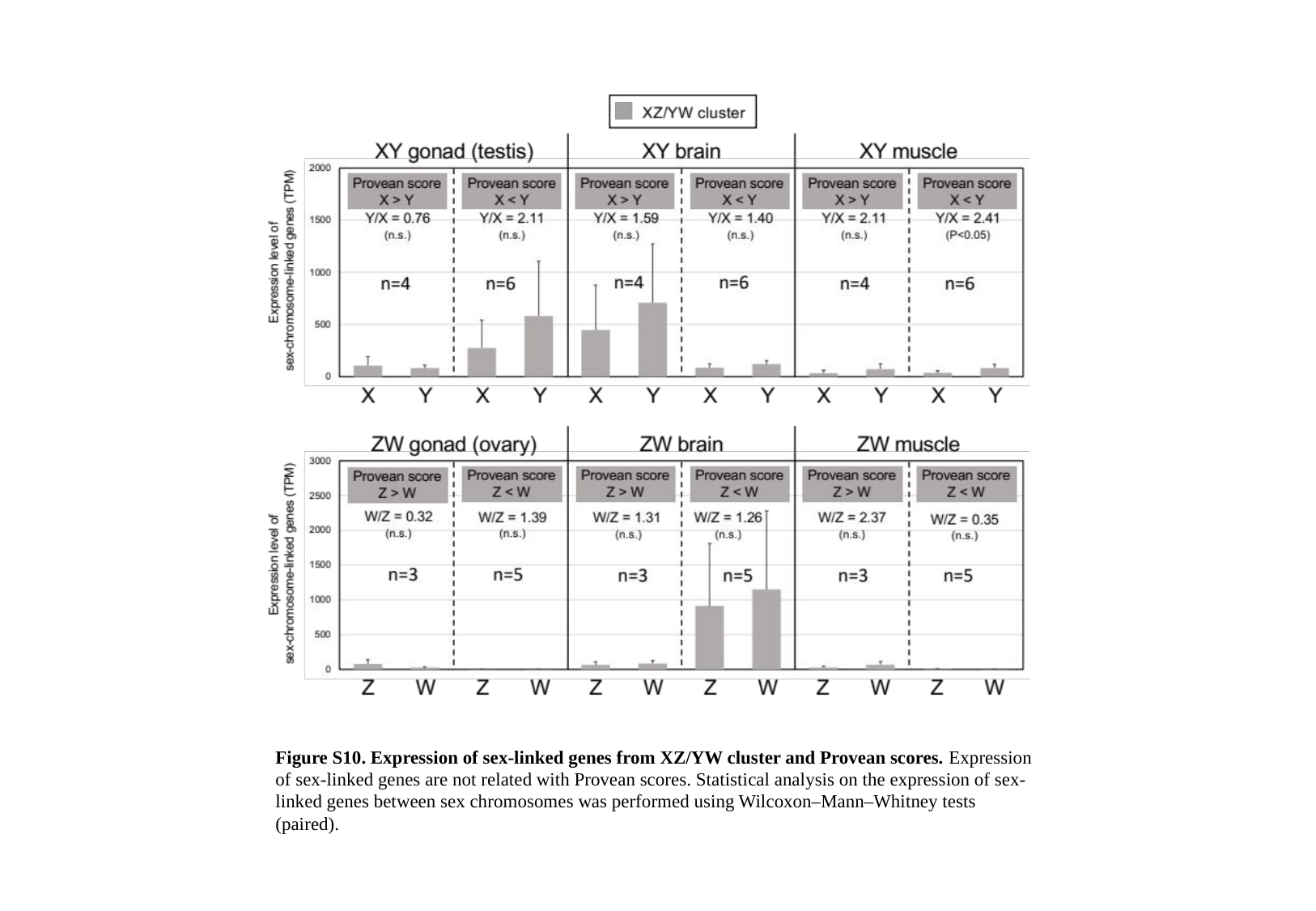

Figure S10. Expression of sex-linked genes from XZ/YW cluster and Provean scores. Expression of sex-linked genes are not related with Provean scores. Statistical analysis on the expression of sex-linked genes between sex chromosomes was performed using Wilcoxon–Mann–Whitney tests (paired).
